# WalkingPad protocol: a randomized clinical trial of behavioral and motivational intervention added to smartphone-enabled supervised home-based exercise in patients with peripheral arterial disease and intermittent claudication

**DOI:** 10.1186/s13063-022-06279-9

**Published:** 2022-04-18

**Authors:** Ivone Silva, Susana Pedras, Rafaela Oliveira, Carlos Veiga, Hugo Paredes

**Affiliations:** 1 Angiology & Vascular Surgery Department, Centro Hospitalar Universitário Porto (CHUP), Porto, Portugal; 2 INESC TEC and Universidade de Trás-os-Montes e Alto Douro (UTAD), Vila Real, Portugal

**Keywords:** Peripheral arterial disease, Intermittent claudication, Home-based exercise therapy, Behavioral change and motivational intervention, Randomized clinical trial, Protocol, m-health

## Abstract

**Background:**

Physical exercise is a first-line treatment for peripheral arterial disease (PAD) and intermittent claudication (IC) reducing pain and increasing the distances walked. Home-based exercise therapy (HBET) has the advantage of reaching a higher number of patients and increasing adherence to physical exercise as it is performed in the patient’s residential area and does not have the time, cost, and access restrictions of supervised exercise therapy (SET) implemented in a clinical setting. Even so, rates of adherence to physical exercise are relatively low, and therefore, m-health tools are promising in increasing motivation to behavior change and adherence to physical exercise. A built-in virtual assistant is a patient-focused tool available in a mobile interface, providing a variety of functions including health education, motivation, and implementation of behavior change techniques.

**Methods:**

This is a single-center, prospective, three-arm, single-blind, randomized, controlled, superior clinical trial with stratified and blocked random allocation. Three hundred participants with PAD and IC will be recruited from an Angiology and Vascular Surgery Department, Centro Hospitalar Universitário Porto (CHUPorto), Porto, Portugal. All patients will receive the same medical care recommended by  current guidelines. Participants in all three groups will receive a personalized prescription for an HBET program and a behavioral change and motivational intervention. Participants in experimental groups 1 and 2 will receive a smartphone with the WalkingPad app to monitor exercise sessions. Experimental group 2 WalkingPad app will have a built-in virtual assistant that will promote behavioral change and provide motivational support. Participants allocated to the active control group will not receive the m-health tool, but a practice diary to encourage monitoring. The  program will last for 6 months with three evaluation moments (baseline, 3, and 6 months). The primary outcome will be the change in distances walked (maximal and pain-free) from baseline to 3 and 6 months. Secondary outcomes will be changes in quality of life, patients’ perception of resistance, and walking speed.

**Discussion:**

This study will allow measuring the effectiveness of an m-health tool in increasing motivation for behavior change and adherence to an HBET program in patients with PAD. The superiority of experimental group 2 in the primary and secondary outcomes will indicate that the virtual assistant is effective for motivating behavioral change and encouraging the practice and adherence to physical exercise. The use of m-health tools and virtual health assistants can potentially fill a gap in the access and quality of health services and information, reducing the burden on the health system and promoting self-management and self-care in chronic illness.

**Trial registration:**

ClinicalTrials.govNCT04749732. Registered on 10 February 2021

**Supplementary Information:**

The online version contains supplementary material available at 10.1186/s13063-022-06279-9.

## Administrative information

Note: the numbers in curly brackets in this protocol refer to SPIRIT checklist item numbers. The order of the items has been modified to group similar items (see http://www.equator-network.org/reporting-guidelines/spirit-2013-statement-defining-standard-protocol-items-for-clinical-trials/).

**Table:** 

Title {1}	WalkingPad protocol: a randomized clinical trial of behavioral and motivational intervention added to smartphone-enabled supervised home-based exercise in patients with peripheral arterial disease and intermittent claudication
Trial registration {2a and 2b}.	The protocol was registered on the U.S. National Library of Medicine (ClinicalTrials.gov) with the identifier NCT04749732 on 10th February 2021. WHO trial Registration Data Set link https://trialsearch.who.int/Trial2.aspx?TrialID=NCT04749732
Protocol version {3}	This is version 2.0 of the protocol, March 9, 2022.
Funding {4}	This study is financed by the FEDER - European Regional Development Fund through NORTE 2020 - Northern Regional Operational Program, under PORTUGAL 2020 and by national funds, through the FCT - Foundation for Science and Technology, within the scope of the project with the reference NORTE-01-0145-FEDER-031161- PTDC / MEC-VAS / 31161/2017; The funding agency played no role in the study design, data collection, analysis, and interpretation of data or in the writing of the manuscript.
Author details {5a}	**Authors**: Ivone Silva^1^, Susana Pedras^2^, Rafaela Oliveira^3^, Carlos Veiga^4^ & Hugo Paredes^5^ ^**1**^ **Ivone Silva**, PhD in Medical Sciences, Angiology & Vascular Surgery Department, Centro Hospitalar Universitário Porto (CHUP), Porto, Portugal Email: heitor.ivone@gmail.com ORCID https://orcid.org/0000-0002-3875-5279 ^2^**Susana Pedras,** PhD in Applied Psychology, Angiology & Vascular Surgery Department, Centro Hospitalar Universitário Porto (CHUP), Porto, Portugal Email:susanapedras@gmail.com ORCID: https://orcid.org/0000-0001-5771-562X ^3^ **Rafaela Oliveira**, Bachelor in Sciences, Angiology & Vascular Surgery Department, Centro Hospitalar Universitário Porto (CHUP), Porto, Portugal Email: rafaela.oliveira.37@outlook.pt ^4^**Carlos Veiga**, Master in Medicine, Angiology & Vascular Surgery Department, Centro Hospitalar Universitário Porto (CHUP), Porto, Portugal Email: carlosdfveiga@gmail.com ORCID: https://orcid.org/0000-0001-8721-0702 ^5^**Hugo Paredes**, PhD in Computer Science, Engineering Department of School of Sciences and Technology, Universidade de Trás-os-Montes e Alto Douro: Universidade de Tras-os-Montes e Alto Douro Email: hparedes@utad.pt ORCID: https://orcid.org/0000-0002-4274-4783
Name and contact information for the trial sponsor {5b}	Angiology & Vascular Surgery Department of Centro Hospitalar Universitário Porto (CHUPorto), Porto 4099-001, Portugal Contact: (+351) 22 207 7500 Webpage: https://www.chporto.pt/
Role of sponsor {5c}	The sponsor played no part in study design; data collection, management, analysis, and interpretation of data; writing of the report; and the decision to submit the report for publication.

## Introduction

### Background and rationale {6a}

Cardiovascular (CV) disease remains the leading cause of death worldwide with the 2013 Global Burden of Disease study estimating that it accounted for 31.5% of all deaths globally [1], representing a considerable economic burden to society and healthcare systems. Lower extremity peripheral artery disease (PAD) is a common cardiovascular disease that affects 27 million people in Europe and the USA. Intermittent claudication (IC) is usually the first symptom of PAD, resulting in walking impairment due to muscle ischemia. IC is defined as fatigue, discomfort, cramping, or pain of vascular origin in the muscles of the lower extremities that is consistently induced by exercise (same distance) and consistently relieved by rest [2]. Severity is generally described in terms of claudication distance, and it can be objectively measured by treadmill test with the assessment of pain-free walking distance (PFWD), maximal walking distance (MWD), and functional walking distance (FWD). However, recent evidence states that the 6-min walk test assessed in a 30-m walkway is a more valuable measure as it is more representative of daily life walking [3]. Furthermore, the discomfort related to IC contributes to a sedentary lifestyle, decreasing physical fitness level, enhancing cardiovascular risk factors, and increasing rates of mobility loss, thus leading to disease deterioration. Hence, the significant functional decline in patients with PAD is associated with the severity of IC, which, in turn, is correlated with higher morbidity, reduced quality of life (QoL), and increased mortality [4–6].

Thus, exercise therapy programs have been recommended as first-line therapy for patients with PAD and IC [7], improving walking ability, functional status, and health-related QoL. Data from randomized clinical trials suggests that exercise therapy is the best initial treatment for IC and show improvements in PFWD and overall walking performance [8, 9]. Regular aerobic exercise also reduces cardiovascular risk and increases the event-free survival rate from 57 to 81% in patients with PAD [10]. In addition, walking distance can decrease by 8.4 m per year after the onset of IC [11]. Therefore, exercise therapy can break this cycle and even reverse it, being an effective, low-cost, and low-risk option compared to more invasive therapies for IC.

Supervised exercise therapy (SET) is the gold standard for walking therapy for patients with PAD. SET programs take place in a hospital or outpatient facilities, in which intermittent walking exercise is used as the treatment modality and is directly supervised by qualified healthcare providers. The current American College of Cardiology/American Heart Association (ACC/AHA) guidelines recommend SET for the treatment of IC as a class I level A recommendation [2, 12]. These guidelines suggest that exercise training, in the form of walking, should be performed for a minimum of 30 to 45 min per session, 3 to 4 times per week, for a period not less than 12 weeks. Although SET programs offer proven benefits, they remain an underutilized tool and inaccessible for most patients. Furthermore, effectiveness is generally limited by poor patient adherence as around 30% simply refuse to participate in these exercise-training programs [12]. Widespread implementation of SET is often also constrained by a lack of facilities and funding [12–14].

Home-based exercise therapy (HBET) is a structured, unsupervised, self-directed program that takes place in the personal setting of the patient rather than in a clinical setting. HBET programs are self-directed with the guidance of healthcare providers who prescribe an exercise plan in all similar to SET programs [13]. However, HBET program implementation is more feasible and eliminates conditioning barriers and impediments such as transportation issues, proximity to clinics, costs and conflicts with the job, and occupational responsibilities. Even though HBET has shown to be effective at improving walking performance and distance, its´ results fall below those seen in SET [14–17]. A 2018 Cochrane systematic review listed moderate- and high-quality evidence that SET improves walking distance to a greater extent than HBET, with results at 3 months showing increases in MWD of 120 to 210 m higher, favoring SET over HBET [18]. Furthermore, current ACC/AHA guidelines give HBET a class II level B recommendation [2] and the European Society of Cardiology/European Society of Vascular Surgery (ESC/ESVS) 2017 guidelines recommend unsupervised exercise training only when SET is not feasible or available [7]. Nevertheless, HBET programs may not only reach a greater number of PAD patients receiving exercise therapy but may also play an important role in promoting continued exercise (behavior maintenance) after participation in SET programs, improving the long-term results.

Successful HBET programs include patient education, exercise self-monitoring, and behavioral intervention that promote adherence to the exercise prescription/plan [17, 18]. Using a behavior change approach is crucial to encourage the patient to initiate, sustain, and progress through the program [15–17]. However, in the last decade, several strategies have been studied and proposed by the scientific and medical community to ensure the regular practice of physical activity by patients with PAD [19–22]. Nevertheless, the results fall short of what is desirable in relation to adherence to physical activity. Thus, innovative home walking programs need to be developed to improve results (behavior change and adherence to physical exercise) and reach a greater number of patients with PAD.

Recently, the use of technology has gained space in this domain. Modern smartphones containing GPS sensors are widely used in our daily life. A dedicated smartphone application may be a valid alternative for measuring walking behavior. Such technologies may improve monitoring, adherence, and consequently clinical outcomes of exercise therapy in patients with PAD [23, 24]. Information and communication technology (ICT) tools have already been applied in patient monitoring during HBET programs, such as geographical information system (GIS) tools (such as GoogleMaps), which are home-based, inexpensive, and easy-to-use tools with a wide range of availability. A recent study using GoogleMaps concluded that it can provide enough accuracy for monitoring PAD home-based exercise, delivering a landmark-based method that objectively measures patients’  adherence to an exercise plan [24]. GoogleMaps may give us a truer representation of walking assessment in patients with IC as it incorporates real-world walking environments. In addition, the use of activity monitoring with accelerometers, pedometers, or even self-report diaries is advisable as it provides the patient with tangible feedback on progress, improving adherence to HBET programs [19–21, 23]. Thus, the monitoring options in HBET can be extended by the incorporation of electronic health (e-health) and mobile health (m-health) technologies [25, 26].

Three randomized trials of 493 patients with PAD demonstrated that home walking exercise interventions that incorporate behavior change techniques improved walking ability and improved 6-min walk test performance more than exercise interventions supervised on treadmill (45–54 m vs. 33–35 m) [27]. Therefore, the use of m-health tools to provide virtual training, goal setting, and reward systems (among others) through a smartphone application proves to be an extremely promising option to promote exercise therapy, above all, to assist in long-term behavioral change, which is a major challenge for programs that require behavioral change [28]. But recently, built-in virtual assistants have been developed to help individuals manage and deal with various types of chronic illnesses. These virtual assistants have the potential to provide advice on chronic illness and self-care, as well as provide health education and encourage behavior change [29–32].

Therefore, research is needed to explore ways to optimize components of HBET programs to improve outcomes and make exercise therapy inexpensive, motivating, and widely accessible to a greater percentage of patients with PAD. Thus, we expect that HBET programs for patients with PAD, supported by an m-health tool with a built-in virtual assistant, will mitigate high dropout rates from programs and encourage adherence to exercise therapy in a patient-chosen setting. Thus, this project will develop and implement an m-health tool that will (1) monitor and provide personalized feedback during and after physical exercise; (2) provide behavior change strategies tailored to individual performance, skills, and needs; and (3) use gamification strategies to increase motivation through fun, promoting self-efficacy and perseverance. This set of elements will be effective in promoting and maintaining long-term adherence to physical exercise therapy.

### Objectives {7}

The purpose of this randomized controlled trial is to evaluate the effectiveness of a HBET program prescribed by healthcare professionals as a treatment for PAD, along with a behavioral change and motivational intervention to promote exercise adherence which, in turn, will improve MWD, PFWD, and FWD, in patients with PAD and IC. Our hypothesis is that the participants who will use the WalkingPad app with a built-in virtual assistant will show greater adherence to physical exercise and, consequently, will improve the distances walked (MWD, PFWD, and FWD) and walking abilities (distance, speed, climbing stairs), when compared with participants who will use the WalkingPad app with tracking and feedback functionality, and those who will not receive the m-health tool.

### Trial design {8}

The WalkingPad protocol is a single-center, prospective, three-arm, single-blinded (to patients) randomized controlled, and a superior clinical trial with blocked and stratified random allocation.

## Methods: participants, interventions, and outcomes

### Study setting {9}

The Standard Protocol Items: Recommendations for Intervention Trials (SPIRIT) 2013 checklist that supports this study can be consulted in Additional file 1. The study will be conducted in the Angiology & Vascular Surgery Department of Centro Hospitalar Universitário do Porto (CHUPorto), Porto, Portugal.

### Eligibility criteria {10}

Inclusion criteria will be the following: (1) PAD with IC (Fontaine II or Rutherford 1–3) due to atherosclerotic disease, (2) Ankle Brachial Index below 0.9 at rest or below 0.73 after exercise (20% decrease), (3) age range between 50 and 80 years, and (4) MWD in treadmill test between 50 and 500 m.

Exclusion criteria will be the following: (1) asymptomatic PAD; (2) critical ischemia (Fontaine III/IV or Rutherford 4–6); (3) previous lower extremity vascular surgery, angioplasty, or lumbar sympathectomy; (4) any condition other than PAD that limits walking; (5) unstable angina or myocardial infarction diagnosed in the last 6 months; (6) inability to obtain ABI measure due to non-compressible vessels; (7) use of cilostazol and pentoxifylline initiated within 3 months before the investigation; (8) active cancer, renal disease, or liver disease; (9) severe chronic obstructive pulmonary disease (GOLD stage III/IV); (10) severe congestive heart failure (NYHA class III/IV); (11) diagnosis of a psychiatric disease that impairs daily life activities and/or with medical records of decompensation episodes in the last year and/or non-adherence to drug therapy; and (12) cognitive impairment (MMSE ≤ 15 for illiterate patients, 22 for those with 1–11 years of schooling; 27 for > 11 years). The program will be implemented by a research team composed of the following elements: vascular surgeons, a nurse, a health psychologist, a clinical physiologist technician, and computer engineers.

### Who will take informed consent? {26a}

The vascular surgeon responsible for identifying participants who meet the inclusion criteria will invite them to participate in the study and obtain written informed consent at the first hospital visit.

### Additional consent provisions for collection and use of participant data and biological specimens {26b}

On the consent form, participants will be asked whether they agree to the use of their data if they choose to withdraw from the trial. Participants will also be asked for permission for the research team to share relevant data with people from the Universities participating in the research or regulatory authorities, where relevant. This trial does not involve the collection of biological specimens for storage.

## Interventions

### Explanation for the choice of comparators {6b}

HBET is not as effective as SET in increasing walking abilities and distances, but on the other hand, it can reach a larger number of patients and increase adherence to exercise therapy not only in the short but also in the long term, as it is developed in the individual’s residential environment and not in clinical settings, and it is practical and inexpensive and involves only the free choice of individuals and time management. However, a fundamental element for adherence to physical exercise is motivation. Intrinsic motivation is promoted through behavioral and motivational change strategies that enhance the individual’s self-regulation skills, considering their values and beliefs, and their idiosyncrasies. For these reasons, a behavioral and motivational change intervention will be implemented. In addition, an m-health tool will be developed that will include an application with an in-built virtual assistant, reducing the need for hospital visits and providing various forms of health care [25–28]. This design will allow us to understand which strategy is more effective in promoting adherence to exercise therapy in the short and long-term, whether it is a face-to-face contact (ACG), a mobile application that allows monitoring and feedback adapted to the characteristics of the patient (ExpG1) or a mobile application with an in-built virtual assistant (ExpG2).

### Intervention description {11a}

#### Active control group: WalkingPad group—PaperWPad group

Active control group (ACG) participants will not receive the smartphone with the WalkingPad app. Participants assigned to this group will receive a 24-week walking program supported by a printed HBET prescription. This prescription consists of a walking plan lasting more than 30 min per session, frequency of at least three sessions per week, and use of near-maximal pain during training as an outcome of claudicating pain. This group will receive an education component on PAD, a behavioral change and motivational intervention, in two face-to-face sessions facilitated by a health psychologist. In addition, participants will receive reinforcement phone calls throughout the 24 week of the study. A self-fulfilling walking logbook will be given to patients to promote self-monitoring. The logbook has the following indicators: date and time, duration of the walk, number, and reasons for stopping, and a question that assesses self-satisfaction after the walk [on a scale from 0 (not at all) to 10 (very satisfied)].

#### Experimental group 1: WalkingPad plus psychological intervention—PsyWPad group

Participants in experimental group 1 (ExpG1) will receive an HBET prescription consisting of a walking plan lasting more than 30 min per session, frequency of at least three sessions per week, use of near-maximal pain during training as an outcome of claudicating pain. This group, like the ACG, will receive an education component on PAD, a behavioral change and motivational intervention, in two face-to-face sessions facilitated by a health psychologist. In addition, reinforcement telephone calls will be provided throughout the program. The 24-week walking program will be monitored by the WalkingPad app, which will accompany patients on their walks and record their adherence to the program. The application has 3 features that allow to (1) create a personalized walking plan to be applied in the patient’s area of residence, monitoring their performance (frequency, duration and stops); (2) view the weekly progress of the walks, including distance, meters, and steps taken in each of the walks; and (3) assess the presence of pain during the walk, answering yes or no (differentiating feature from all other apps available on the market). Details about the WalkingPad app will be described elsewhere (WalkingPad app usability on paper).

#### Experimental group 2: WalkingPad plus virtual assistant—CyberWPad group

Participants in experimental group 2 (ExpG2) will receive an HBET prescription consisting of a walking plan lasting more than 30 min per session, frequency of at least three sessions per week, use of near-maximal pain during training as an endpoint of claudication pain. In this group, behavior change and motivational intervention will be facilitated by a virtual assistant. This app has the same functionality as the WalkingPad app used on the ExpG1 but includes a virtual assistant that will provide programmed and personalized behavior change techniques and initiate dialogues and send notifications and motivational SMSs [29–33]. The intervention provided through the virtual assistant included in the app will be similar to what participants in the other two groups received in a face-to-face consultation with a psychologist.

The three groups will receive the same behavioral change and motivational intervention based on the principles of the theoretical framework of the Self-Determination Theory (SDT). ACG and ExpG1 will receive face-to-face intervention and telephone support during the study, taking into account the components of the Transtheoretical Model of Change (TTMC) and the Theory of Planned Behavior (TPB). ExpG2 participants will receive a conceptually similar intervention, but through interactions with a virtual assistant, without face-to-face or telephone contacts.

### Criteria for discontinuing or modifying allocated interventions {11b}

There will be no special criteria for discontinuing or modifying allocated interventions. Interventions will not be modified but will be discontinued at the request of the participant or due to worsening PAD symptoms or other pre-morbid conditions. Participants will be aware that participation is voluntary and that they can withdraw at any time. However, whenever possible, information on the main reason(s) for withdrawal will be requested and recorded for further analysis in the interpretation of results.

### Strategies to improve adherence to interventions {11c}

Strategies to improve adherence to the intervention protocol will be as follows: (a) reinforcement telephone calls will be made as part of the theoretical approach to behavior change adopted in this study but which will also work as a retention strategy; (b) at time 2 (at 3 months) and at time 3 (at 6 months), two medals (pins) with the project symbol will be offered (beginner WalkingPad member and advanced WalkingPad member) and a personalized report with the systematization of the progress achieved over time; (c) a WalkingPad technical support phone number will be provided for experimental groups in case of difficulties connecting to the app and a written instruction book will be provided with step-by-step help information for dealing with the app, thus avoiding, withdrawal due to difficulties in working with the app.

The following measures of feasibility will be considered: (1) recruitment rates (e.g., the proportion of subjects who responded with interest to our invitation; the proportion of subjects who agreed to participate in the study, regardless of eligibility; the proportion of subjects who completed the first evaluation and started the intervention); (2) dropout rate (e.g., percentage of participants who did not complete the program (the three assessments over 24 weeks); (3) adherence rate (the number of exercise sessions per week during the 24 weeks must be ≥ to 3 per week); and (4) completion rate (percentage of participants who completed the program).

### Relevant concomitant care permitted or prohibited during the trial {11d}

Implementation of the WalkingPad program will not require a change in usual care pathways (including the use of any medication) in the three arms of the study. Thus, there will be no restrictions regarding concomitant care during the trial.

### Provisions for post-trial care {30}

There is no anticipated harm and compensation for participating in the trial. However, post-trial care encompasses the following responsibilities: referring patients for appropriate follow-up care (other medical specialty or counseling) or providing alternative therapeutic measures (change of medication or consideration of surgery).

### Outcomes {12}

#### Primary outcome

The primary endpoint will be the WalkingPad-induced change in PFWD, FWD, and MWD measured using a standardized treadmill test and the 6-min walk test (6MWT). PFWD is defined as the distance walked at the onset of claudication pain. FWD is defined as the distance at which a patient chooses to stop because of the discomfort caused by claudicating pain. MWD is defined as the distance at which claudication pain becomes so intense that the patient is forced to stop.

#### Secondary outcomes

Secondary outcomes include the change in general and specific quality of life questionnaires and in the patient’s perceived walking distance, speed, and ability to climb stairs.

#### Behavioral process variables

Process variables are those that can influence and explain initial and behavioral change over time. These psychological variables will play an important role in the change process as initial predictors in explaining the onset of behavioral change and as mediators and/or moderators in explaining the maintenance of change.

#### Additional variables

Sociodemographic, clinical, and physical data will be collected from the electronic medical record of the participants, through self-report and clinical interview. Data on physical capacity will be collected through two tests (treadmill test and 6-min test).

### Participant timeline {13}

**Fig. 1:**
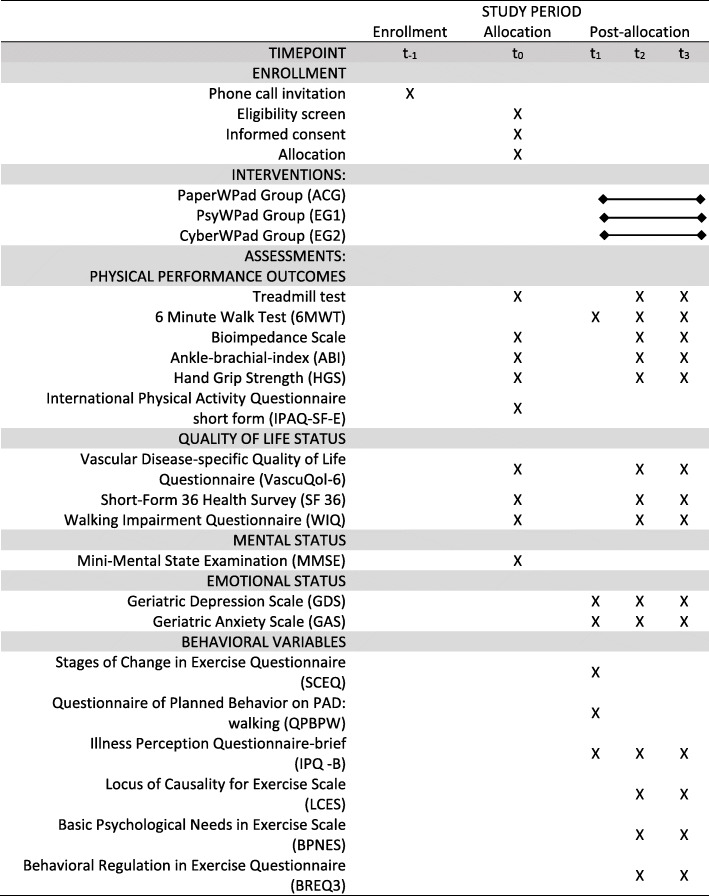
Schedule of enrollment, interventions, and assessments

Time 0 (t0) will be performed as a screening evaluation to ascertain inclusion criteria. Measurements will take place at time 1 (t1 - baseline assessment), 3 months after t1 (time 2: t2), and 6 months after t1 (time 3: t3). The schedule of enrolment, interventions, and assessments is shown in Fig. 1. Participants’ recruitment has already begun.

### Sample size {14}

The sample size was calculated for an effect size of 0.25, a statistical power of 80%, and a significance level of 5%; á priori 57 participants will be needed in each of the groups (G*Power software, version 3.1.9). This calculus was based on previous studies (*F* tests, ANOVA: repeated measures, within-between interaction) [15, 17]. The calculated sample size is expected to be reached by the end of May 2022.

### Recruitment {15}

Three hundred patients with PAD and IC, aged between 50 and 80 years, and with a MWD between 50 and 500 m, will be identified and recruited from the outpatient database of the Angiology & Vascular Surgery Department, Centro Hospitalar Universitário Porto (CHUPorto), Porto, Portugal, by a vascular surgeon member of the team. Eligibility for the study will be tested using clinical medical records and a screening evaluation. The listed participants will be contacted by phone and invited to participate in the study. The invitation will include a brief description of the study and the benefits of participating. For those who show interest in participating, a clinical, physical, and psychological screening assessment will be scheduled at the hospital—time 0 (t0 before allocation). In this assessment (t0), participants will sign a written consent and will be assessed (1 h ±) to determine particular and specific eligibility criteria. If there is an impediment to participation (e.g., COVID-19), the patient will be contacted later.

## Assignment of interventions: allocation

### Sequence generation {16a}

After the identification of patients through consultation of the outpatient database, the fulfillment of the inclusion criteria in the screening session and the patient’s agreement to participate in the study, a random sequence will be created using a random number allocation software, by a researcher external to the team involved in the execution of this study, in order to guarantee the concealment of the allocation of participants by the different groups. Randomization will be carried out in blocks with four strata and the eligible participants will be randomly assigned to three groups, in blocks of multiples of five, and will be stratified according to (i) age (50 to 65 years old; 66 to 80 years old) and (ii) MWD at the baseline (50 to 275 m; 276 to 500 m). The entire process will be overseen by a researcher who will not be directly involved in the intervention or assessments. This researcher will inform the research team of the final allocation of each participant.

### Concealment mechanism {16b}

Participants will be grouped according to their demographic characteristics (age) and physical performance (walking ability) after screening assessment (time 0). Each participant will be assigned a random number, generated in a computer spreadsheet (Excel sheet). Then, randomization will be performed sequentially by three groups taking into account age and physical performance subgroups. To ensure that participants are assessed (initial screening) and randomized by their initial differentiating characteristics (age and physical performance), allocation concealment will be ensured until all baseline measurements are completed.

### Implementation {16c}

The researchers (vascular surgeons and research assistants) will be responsible for the identification, selection, enrollment, and screening assessment of patients. Prior to the formal assessment, the data analyst will generate the allocation sequence and assign participants to groups. Other researchers will evaluate and implement the interventions (not blinded). The data analyst will analyze the data without uncovering the allocation of patients.

## Assignment of interventions: blinding

### Who will be blinded {17a}

Participants will be blinded to the group they are assigned to; they will only know that all participants will receive a personalized and self-defined walking plan tailored to each one (a physical exercise prescription to be carried out in the area of residence). The researchers (vascular surgeons and research assistants) responsible for identifying, selecting, enrolling, and screening patients will be blinded to group assignments. A data analyst will generate the allocation sequence and assign participants to groups and will be responsible for data analysis. The researcher who will carry out the psychological intervention, the technician who will carry out the physical assessment, and the computer engineer who will define the route of the walking plan tailored to the patient, on the WalkingPad web platform, will not be blind as to the assignment group because each intervention has its specificity. Thus, this will be a single-blind study, as it will not be possible to blind the patients to the outcome or the researchers who implement and administer the intervention.

### Procedure for unblinding if needed {17b}

The design is open-label with only outcome assessors and data analysts being blinded, so unblinding will not occur.

## Data collection and management

### Plans for assessment and collection of outcomes {18a}

#### Data collection instruments for the primary outcome

**Fig. 2:**
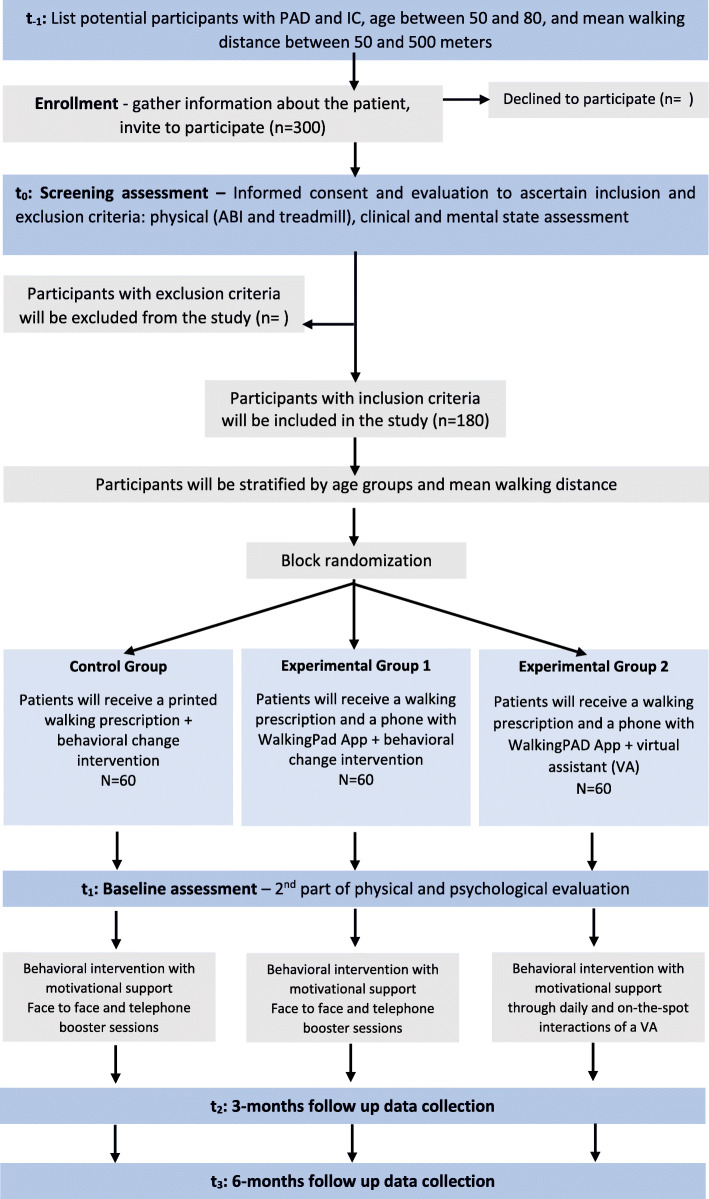
Flow diagram of the clinical trial

The primary and secondary outcomes will be collected via self-report measures and through physical performance tests which are briefly described in Table 1. The timing of these measurements is shown in Fig. 2.

**Table 1:** Summary of self-report primary and secondary outcomes, process, and screening variables, and respective assessment measures

Outcome	Measure	Brief description
Primary		
MWD PFWD FWD	*Treadmill test (TT)*	The treadmill test is an accepted method used in patients with IC to evaluate walking ability [34]. Performing a treadmill test before and after an intervention can provide an objective assessment of change in performance [35]. The modified Gardner-Skinner Treadmill Protocol will be used, where participants will begin to walk on the treadmill at 1 km/h with a 0% grade. After 2 min, the speed is increased to 1.6 km/h, at 0% grade. Then, the speed is increased by 0.8 km/h every 2 min until reaching 3.2 km/h. After reaching 3.2 km/h, the speed is kept constant, and the grade is increased by 2% every 2 min. The main difference between the modified protocol used and the original Gardner-Skinner Treadmill Protocol [36] is that the protocol used is better adapted to this study sample capabilities and equipment, since the original Gardner-Skinner Treadmill Protocol has a continuous speed of 3.2 km/h, and the grade is increased by 2% every 2 min. During the exercise, blood pressure and heart rate will be continuously monitored (Domyus Incline Rune, China). To implement the treadmill protocol, patients must be at rest, informed about the termination criteria (excluding safety criteria), about permission to use the handrail support, and informed about the claudication pain scale used [37]. The termination criterion is the limit of 500 m of pain-free walking distance (PFWD), but there are additional endpoints: voluntary exhaustion, fatigue and shortness of breath, and severe pain in another part of the body (e.g., spine). The claudication pain scale is a continuous scale from 0—indicating no pain, to 4—indicating severe pain, and patients are instructed to walk to near-maximal pain levels [37]. No encouragements will be given during the test. The test administrator has the necessary qualifications and is familiar with the protocol.
PFWD	*6-min walk test (6MWT)*	The 6MWT is a performance-based measure that evaluates the functional capacity of the individual to walk over a total of 6 min on a 100 ft (≈30 m) hallway, providing information regarding all the systems during physical activity [35]. The participants will be instructed to walk back and forth along the hallway to achieve the greatest distance possible. The participants will be allowed to stop and rest while the stopwatch continues to run. The American Thoracic Society guidelines will be used [38]. No encouragement through standardized verb phrases will be given because the aim is for the test to be as similar as possible to what happens in a real environment.
**Secondary outcomes**	**Measure**	**Brief description**
Physical and Mental Quality of Life	*Short-Form Health Survey (SF-36)*	This instrument consists of 36 items with different response scales assessing eight health concepts: limitations in physical activities because of health problems,limitations in social activities because of physical or emotional problems, limitations in usual role activities because of physical health problems, bodily pain, general mental health (psychological distress and well-being), limitations in usual role activities because of emotional problems, vitality (energy and fatigue), and general health perceptions. The SF-36 has been widely used in studies with this population and has excellent psychometrics [39, 40].
Vascular Disease-specific Quality of Life	*Vascular Disease-Specific Quality of Life Questionnaire (VAscuQoL-6)*	This is a specific measure of health-related QoL for patients with PAD, consisting of six items with different response scales [41, 42]. The total score ranges from 6 to 24, with higher results corresponding to a higer quality of life associated with arterial disease. The Brazilian-Portuguese version of the VascuQoL-6 presents adequate valid and reliable indicators allowing its use in patients with PAD with intermittent claudication symptoms [43].
Walking difficulties	*Walking Impairment Questionnaire (WIQ)*	This instrument assesses walking performance/abilities in three domains: distance (distances the individual can walk), speed (the speed the individual can walk), and stairs (number of stairs that the individual can climb without stopping), in a 5-point Likert scale (“none, slight, some, quite difficult, unable”). The distance comprises 7 items with a total score ranging from 0 to 28, with the highest results corresponding to a greater walked distance; speed has 4 items with a total score ranging from 0 to 16, with higher values indicating greater speed; stairs contain 3 items with a total score ranging from 0 to 12, with higher results indicating a greater ability to climb stairs [44]. The Brazilian-Portuguese version of the WIQ showed significant correlations between the WIQ domains and the SF-36 (functional capacity, physical aspects, bodily pain, and emotional aspects) and physical fitness performance (treadmill and strength tests). Intraclass coefficient correlation ranged from 0.72 to 0.81, and there were no differences in WIQ scores between the two questionnaire applications [45].
**Process variables**	**Measure**	**Brief description**
Illness representations	*Illness Perception Questionnaire - Brief (IPQ-B)*	This questionnaire contains 8 items assessing the cognitive and emotional representations of the disease in eight specific dimensions: consequences, timeline, personal control, treatment control, identity, concerns, understanding, and emotional representations [46, 47]. The response scale for each item ranges from 0 to 10 and higher scores indicate more threatening perceptions in each item/dimension concerning PAD. The Portuguese version showed good internal consistency [48]. This instrument has already been used in studies with this population.
Motivation stage for the change	*Stages of Change in Exercise Questionnaire (SCEQ)*	This questionnaire is composed of five items that represent each of the five stages of the Transtheoretical Model [49]. The Pre-Contemplation stage is characterized by the absence of intention to change behavior in the next 6 months (e.g., *I do not walk, and I do NOT intend to start walking in the next 6 months*). Contemplation is defined as the intention to change behavior within the next 6 months. When the individual intends to initiate behavior change within 1 month, the individual is classified as being in the Readiness to the Action stage. The Action stage is distinguished by having initiated a consistent and continued behavior change for 6 months or more and by moving the individual to the Maintenance stage. Subjects should indicate which item reflects their current exercise behavior in a dichotomous format of yes or no [50–52].
Locus of causality for exercise	*Locus of Causality for Exercise Scale (LCES)*	This scale comprises three items assessing the perceived choice (or autonomy) regarding performing physical exercise. Thus, this scale assesses the extent to which individuals feel that they freely choose to exercise (walking) rather than feeling that they have to for some reason, addressing the source of the initiation of behavior. An internal locus of causality is evident when an individual engages in a behavior freely and with no sense of coercion. The response scale on a 6-point Likert scale ranges from 1 to 6 and the total score ranges from 3 to 18. Higher scores indicate greater self-determination or a more internal perceived locus of causality [53]. The Portuguese version showed good internal consistency [54].
Planned behavior	*Questionnaire of Planned Behavior on PAD-Walking (QPBPW)*	This questionnaire assesses intentions, attitudes, subjective norms, perceived control, action, and coping plans regarding walking, in patients with PAD. The intentions scale is composed of 2 items with scores ranging from 2 to 10 points and higher scores indicating greater intention to perform the exercise (walking). The attitudes scale consists of 5 items, with scores ranging from 5 to 25 points and higher scores indicating a more positive attitude towards exercise. The subjective norms scale is composed of 3 items with scores ranging from 3 to 15 points, in which the higher the score, the higher is the perception of the importance attributed by other people to exercise. The perceived behavioral control scale evaluates the perception of control over-exercise, and it is composed of 4 items, with scores ranging from 4 to 20 points, and higher scores indicating a greater perceived behavioral control. The action and coping planning scale consist of 9 items, with scores ranging from 9 to 45 points, and higher scores indicating more action and coping plans regarding exercise (adapted for walking) [55]. The instrument has good internal consistency and was originally developed to be used with individuals with type 2 diabetes, and in this study, it was adapted to be used with patients with PAD, regarding walking.
Satisfaction of basic psychological needs	*Basic Psychological Needs in Exercise Scale (BPNES)*	This scale consists of 12 items and assesses the perception of satisfaction of the three basic psychological needs in the context of the exercise: autonomy, competence, and positive relationship (relatedness) on a 5-point Likert scale (“strongly disagree” to “strongly agree”). Scores range from 12 to 60 and higher scores indicate a greater perceived satisfaction of psychological needs during exercise [56, 57]. The Portuguese validation was performed on a sample of regular exercise participants and showed good psychometric properties in the three scales. For this study, the instructions were adapted to walking behavior.
Self-regulation in exercise	*Behavioral Regulation in Exercise Questionnaire (BREQ-3)*	This scale has 18 items, divided into six scales, assessing motivational regulations for exercise with a score ranging from 0 to 12 for each type of regulation on a 5-point Likert scale (“strongly disagree” to “strongly agree”). Higher scores indicate higher levels of one of the following types of behavioral regulation: amotivation, external, introjected, identified, integrated, and intrinsic [58–60]. The Portuguese validation was carried out on a sample of gym practitioners and showed good psychometric qualities in the six scales/types of motivation and regulation. For this study, the instructions were adapted to walking behavior.
**Screening measures**	**Measure**	**Brief description**
Sociodemographic and clinical data	*Sociodemographic and clinical data questionnaire*	It consists of information to be obtained directly from participants or clinical records: gender, age, living environment, marital and professional status, rural or urban areas of residence. Clinical data: clinical and surgical history, chronic medication, and lifestyle behaviors (alcohol and tobacco consumption, hours of sleep, and the number of daily meals).
Cognitive status	*Mini-Mental State Examination (MMSE)*	This is a widely used test of cognitive function, including tests of orientation, attention, memory, language, and visual-spatial skills. The total score ranges from 0 to 30, and higher results correspond to a better mental state. It will be applied at baseline as part of the screening assessment to ascertain exclusion criteria [61, 62].
Emotional status	*Geriatric Depression Scale-5 (GDS)* *Geriatric Anxiety Scale (GAS)*	As they are part of the screening, both questionnaires chosen to assess emotional state are very small, with dichotomous response scales, validated for a population over 65 years of age. Depressive symptoms are assessed through 5 items, with scores ranging from 0 to 5, and higher results corresponding to more depressive symptoms [63, 64]. A GDS-5 score ≥ 2 suggests clinical depression and the need for further evaluation. The Portuguese version has acceptable internal consistency. Anxiety symptoms are assessed through five items, with scores ranging from 0 to 5, and higher results corresponding to more anxiety symptoms [65, 66]. Portuguese internal consistency is high and a score ≥ 3 was optimal for the detection of DSM-IV Generalized Anxiety Disorder.
Physical activity	*International Physical Activity Questionnaire for elderly-Short Form (IPAQ-SF-E)*	The version adapted for the elderly was used, as individuals with PAD avoid physical activity due to claudicating pain and it is not expected to find patients practicing high levels of physical activity or regular physical exercise. This version, although validated for people over 65 years old, is smaller and the items are more adapted to the performance level of this sample. Thus, this version consists of 4 self-reported moderate-to-vigorous physical activity (MVPA) and sedentary behavior (sitting) items. The items encompass the following behaviors, in the last 7 days: the time spent sitting, the days and time spent walking, the days and time spent in moderate-intensity activities, and the days and time spent in vigorous-intensity activities. Scores range from 0 to indefinite minutes of physical activity per week and higher results correspond to a greater amount of physical activity performed. Results can be reported in categories (low, moderate, or high activity levels) or as a continuous variable (MET minutes per week). MET minutes represent the amount of energy expended carrying out physical activity [67–69]. The specificity of IPAQ-SF-E to identify low-active participants was 85%, and the sensitivity to identify the more active participants was 81%.
**Physical measures**	**Measure**	**Brief description**
**Physical measures**	*Ankle-brachial-index (ABI)*	Ankle Brachial Index (ABI) is the first low-cost diagnostic test for PAD [2]. The ABI is a simple and non-invasive test that will be obtained before and after the treadmill test by measuring the systolic pressures at the brachial artery, anterior tibial artery, and posterior tibial artery, in the supine position, in millimeters of mercury (mmHg), using a Doppler device. The ABI of each leg will be calculated by dividing the higher mean of three measures of the anterior tibial pressure or posterior tibial pressure by the higher mean of three measures of the right or left arm pressure (LifeDop 150 Doppler (8 MHz), USA).
**Hand strength**	*Hand grip strength (HGS)*	Hand grip strength (HGS) is a basic measure for determining musculoskeletal function, as well as weakness and disability [70]. The HGS produces an isometric strength measure that allows the identification of the muscular weakness of the upper limb and provides an indication of the overall strength since it reflects the strength of the lower limbs. Three consecutive measures of handgrip strength (in kilograms (kg)), at both hands, will be recorded in a standing position with the arms next to the body, elbow slightly flexed and wrist in a neutral position, through a hand-held dynamometer (Gripx EH101, China).
**Body composition**	*Body composition measures*	Weight (in kilograms), body mass index (kg/m^2^), body fat percentage (%), visceral fat level (%), skeletal muscle percentage (%), and resting metabolism (in kilocalories, kcal) will be measured through a bioimpedance scale (OMRON Body Composition Monitor BF511 (HBF-511 T-E/HBF-511B-E, Japan)). Height (in meters) will be measured using a tape measure.

The primary outcomes will be measured by two tests. The walking distance measured with a standardized treadmill test [34, 35] is a widely used tool for functional assessment and monitoring of exercise rehabilitation, where PFWD, FWD, and MWD are objective measures of improvement. In turn, the 6MWT is an individualized test that assesses the submaximal level of functional capacity [35, 38]. The patient chooses the intensity of the exercise and can rest during the test, which better reflects the functional level of exercise practiced in daily physical activities. Therefore, we chose to use both measures (walking distance measured with the treadmill test and walking distance measured with the 6MWT) not only because they complement each other but also because both provide different and valuable information.

The primary outcomes will reflect the effectiveness (beneficial effect) and not the harm (adverse effect) of the intervention. The change achieved in primary outcomes from baseline to 3 and 6 months will be a valid, reproducible, and relevant result for the target population and healthcare professionals. The amount of change achieved will be represented in a continuous variable that will facilitate comparisons and grouping of results across studies, trials, and meta-analyses. Data collection forms can be requested from the responsible investigator.

### Plans to promote participant retention and complete follow-up {18b}

The implementation of strategies to promote adherence and prevent dropout is one of the most important aspects in trials, especially when the few studies reporting dropout rates, have dropout rates ranging from 12 to 27.5% [15–17]. Thus, to minimize attrition and promote adherence, the following strategies will be used: (1) during the invitation phone call, the physician will explain the objectives, benefits, and importance of participating in the study; (2) participants who agree to participate in the study will be offered a Diploma of Commitment where participation is presented as something positive and not accessible to everyone (Congratulations! You have been selected to participate in this study!). The diploma informs that the participant becomes part of a group of people who will undergo physical exercise treatment for PAD, the start and end dates of treatment and study, and the dates of face-to-face sessions at the hospital for evaluations formalities over the 6 months. (3) In these evaluations, a medal will be offered (pin format) and a report with individualized and systematized information on the results obtained with regard to the main result; (4) reminder messages will be sent whenever the date of the formal hospital assessment approaches; (5) congratulatory messages will be sent on each participant’s birthday, as well as congratulatory messages for being part of the study each month; (6) a Facebook page will be created to share information about the disease and the importance of physical exercise, and (7) a 24-h telephone number will be provided to support participants in the event of adverse events. Participants who discontinue or deviate from intervention protocols will be invited to attend the assessments in order to implement an intention-to-treat data analysis protocol at the end of the study.

### Data management {19}

Data will be entered as it is collected. The data collection forms—a set of questionnaires and the respective quotation for each item and total instrument score—will be transferred to the SPSS database. A member of the research team will enter the data and each time the database is closed will do a descriptive analysis of the data to ensure that the range of data values is correct in each variable. The data collection forms used, and their quotation will be available upon request from the principal investigator. Data will be analyzed by a data analyst who will be blinded to participant allocation. Questionnaires will be destroyed once the data is published.

### Confidentiality {27}

The procedures for storing, processing, and protecting personal data will respect the current General Data Protection Regulation (Regulation (EU) 2016/679), Portuguese Law No. 58/2019 of 8 August. Each participant will be assigned a code (unique identification number) in several databases to ensure data independence. All information about the practice of physical exercise obtained through the mobile app will be stored in an online data system platform. The WalkingPad web platform is accessible to team members and patients. As long as the patient remains in the clinical trial, he accepts that the entire research team has access to the data recorded by the WalkingPad web platform. As for the assessment data, only the principal investigator and the data analyst will have access to the complete dataset, which will be stored in different locations and used for research purposes only. Passwords will be used to ensure the protection and security of data (and participants).

### Plans for collection, laboratory evaluation, and storage of biological specimens for genetic or molecular analysis in this trial/future use {33}

This trial does not involve collecting biological specimens for storage.

## Statistical methods

### Statistical methods for primary and secondary outcomes {20a}

The first step in the data analysis plan will be the screening of data to detect errors and missing values, although missing data is not expected in the questionnaires, as the data collection will be carried out in person with a researcher who will guarantee its complete completion. Missing values may be present when a participant withdraw or refuse to participate in the assessment, or missing values in physical tests due to physical limitations or difficulties in performing physical performance tests (e.g., treadmill test). Second, a summary of baseline sociodemographic, clinical, physical, and psychological variables will be provided through descriptive statistics. Third, comparisons will be made between the three groups to examine whether there are differences between the groups at baseline in all variables. As the sample was stratified, differences in fundamental variables such as age and maximal walking distance are not expected to be found. In addition, differences at baseline between participants who will complete the study and those who drop out will be examined. Fourth, primary outcome analyses will be conducted on an intention-to-treat basis, including all participants analyzed as randomized. The primary outcome will be analyzed using multiple regressions with adjustment for MWD, PFWD, and FWD. A mixed ANOVA will be performed to test the mean differences between the three groups (between-subject factor) and over the three measurement points (in-subject factor). Results will be reported as the difference in MWD, PFWD, and FWD from t1 to t2, t2 to t3, and t1 to t3, as well as the mean differences between GE1 and GE2, and ACG with 95% confidence intervals. To determine the minimal clinically important difference (MCID), a change in scores for the primary outcome will be calculated by subtracting the baseline outcome from the t2 (3 months) and t3 (6 months) outcomes for each participant. The least important difference (MID) for improvement and deterioration will be defined by the upper and lower limits of the 95% confidence interval of the mean change in MWD, PFWD, and FWD [71]. Twenty meters was defined as MCID [17]. However, a recent meta-analysis [19] showed significant heterogeneity in the magnitude of effect estimates for MWD (*χ*^2^ = 16.4, *p* = 0.022) and PFWD (*χ*^2^ = 19.6, *p* = 0.003). The mean change (SD) in MWD for patients randomly allocated to home exercise or programs with no exercise plan ranges from 24 (166) to 402 (391) m and PFWD changes range from 21 (81) to 182 (249) m.

Finally, secondary outcomes (walking ability and general and specific health-related QoL) will be similarly analyzed. Mediation and moderation analyses will be performed to examine mechanisms of influence (process variables) as well as Structural Equation Modeling (SEM) that will allow the analyses of complex relationships between variables and test causal relationships explaining initial behavioral change throughout time. Measures of effect size and statistical power (1−*β*) will be presented for all statistical tests performed. All planned tests are two-tailed. Statistical significance will be set at *P* ≤ 0.05. IBM® SPSS® V.26 and IBM® IPSS® Amos™ will be used to perform all data analyses.

### Interim analyses {21b}

There are no anticipated harmful consequences or real risks for the participants involved in this intervention. Thus, interim analyses, discontinuation rules, or criteria for discontinuing or modifying allocated interventions will not be included in the current protocol. Furthermore, all participants will be aware of their voluntary participation and therefore will be informed that they can withdraw from the program at any time.

### Methods for additional analyses (e.g., subgroup analyses) {20b}

An additional analysis will be performed with the subgroup of patients who were infected with SARS-COV-19 during their participation in the study.

### Methods in analysis to handle protocol non-adherence and any statistical methods to handle missing data {20c}

Before the start of the registration of participants, training will be carried out to instruct all researchers in the collection and entry of data and in the implementation of interventions. Timely data entry will allow early detection of problems with missing data (forgetting, overlapping crosses). In order to detect them, a verification process will be implemented for all questionnaires after completion and for all variables after entering the SPSS database.

### Plans to give access to the full protocol, participant-level data, and statistical code {31c}

We will provide the protocol, participant-level data, and statistical code upon request.

## Oversight and monitoring

### Composition of the coordinating center and trial steering committee {5d}

This study does not have a designated coordinating center or study steering committee. The study is a single center and the research team at the hospital where the study will be carried out will be responsible for conducting and monitoring the daily tasks necessary for the execution of the study. This team meets periodically to assess the conduct and progress of this study and ensure compliance with the study protocol. There are also meetings every 3 months with those responsible for coordinating the study (principal investigator (PI) and co-investigator (Co-PI)) to discuss management and financial issues related to the study. To ensure that the protocol is implemented as planned, the PI will be responsible for managing and overseeing the study and providing guidance and administrative support. Weekly, the research assistants will meet to establish short-term objectives, consult the fulfillment of previously established goals, and identify problems or deviations from the plan.

### Composition of the data monitoring committee, its role, and reporting structure {21a}

It will not be necessary to form a data monitoring committee (DMC) to independently assess the safety, scientific validity, and integrity of the clinical trial because this intervention is a low-risk intervention, does not present substantial safety issues, and does not have a double-blind treatment.

### Adverse event reporting and harms {22}

The present intervention carries minimal risks, so we do not expect any serious or severe adverse events. However, over time participants will be contacted and asked about their physical and mental well-being. In addition, participants will have access to a 24-h telephone contact that they can call if needed. If a serious or severe adverse event occurs, the Ethics Committee will be notified.

### Frequency and plans for auditing trial conduct {23}

The need for an audit is not foreseen; however, the training and research center of the hospital where the study will be carried out may audit the study.

### Plans for communicating important protocol amendments to relevant parties (e.g., trial participants, ethical committees) {25}

Changes in the study protocol will first be submitted to the Ethics Committee of the Hospital where the study will be carried out and, after approval, updated accordingly on the clinical trials website.

## Dissemination plans {31a}

The WalkingPad program results dissemination plan includes (1) writing and publishing articles in indexed journals, including the WalkingPad protocol and the article on the effectiveness of the intervention program and the set of variables that contribute to adherence to physical exercise in the long term; (2) present the results of the study to health professionals at scientific conferences and events; (3) disseminate the study results to the participants through a social event organized at the end of the study; (4) share the results with the public and other relevant groups, via the media (TV and newspapers) and social networks (Facebook, Instagram, and Twitter); (5) update the WalkingPad project website with pertinent information [5]; systematize guidelines on m-health interventions to increase the effectiveness of HBET programs not only in PAD but also in cardiovascular disease in general.

## Discussion

PAD represents a considerable economic burden for society and health systems, for patients and families, and therefore, cost-effective treatments are necessary. As SET programs are difficult to implement systematically in all patients whenever needed, new options must emerge, and research has shown that HBET programs are more accessible, effective, appropriate, cheaper, patient-friendly, and enjoyable (in contact with nature) to control IC in patients with PAD. M-health tools are a promising adjuvant strategy as they are practical, easy to use, and cost-free. Virtual assistants have been successfully used to promote adherence to self-care in various chronic diseases but have not yet been used to promote the practice of physical exercise in patients with PAD. Thus, to the best of our knowledge, this will be the first randomized clinical trial evaluating the effectiveness of an HBET program with a behavioral change and motivational intervention delivered by a virtual assistant, to promote adherence to physical exercise and, consequently, improve walking distances in patients with PAD and IC.

The main limitation of this study refers to the low digital literacy of this population. The inclusion of participants regardless of their level of digital literacy, access to digital devices and to Internet, is likely to be a factor influencing the results. However, the goal is to make an app available to the entire population of people with PAD and not just to the youngest and most digitally literate.

The strengths of this study include that participation requires no more consultations than previously scheduled, no engagement with healthcare professionals or researchers (apart from formal study assessments), and interventions do not have serious effects on individuals when exclusion criteria are assured. Communication with the research assistant will be facilitated with an email address and a phone number. Furthermore, as almost all (if not all) individuals use cell phones, these m-health tools and virtual health assistants can potentially fill a gap in access and quality of healthcare services and information available, reducing the burden on the health system and promoting self-management  and self-care in chronic illness.

## Trial status

At the time of submission, this trial was already recruiting participants. This is version 2.0 of the protocol, last changes on March 9, 2022; expected date for completion of recruitment: April 30, 2022; expected date for the end of data collection: October 30, 2022; study end date: November 2022.

## Supplementary Information

**Additional file 1.** SPIRIT 2013 checklist

## Abbreviations

HBET: Home-based exercise therapy
App: Application
CV: Cardiovascular
PAD: Peripheral arterial disease
IC: Intermittent claudication
PFWD: Pain-free walking distance
MWD: Maximal walking distance
FWD: Functional walking distance
QoL: Quality of life
SET: Supervised exercise therapy
ACC/AHA: American College of Cardiology/American Heart Association
ICT: Information and communication technology
GIS: Geographical information system
ACG: Active control group
ExpG1: Experimental group 1
ExpG2: Experimental group 2
ABI: Ankle Brachial Index
MMSE: Mini-Mental State Exam
NYHA: New York Heart Association
SDT: Self-Determination Theory
TTMC: Transtheoretical Model of Change
TPB: Theory of Planned Behavior
6MWT: 6-min walk test
IPQ-B: Illness Perception Questionnaire–Brief
M/RQW: Motivation and Readiness Questionnaire for Walking
SOC: Stage of Change
LCES: Locus of Causality for Exercise Scale
QPBPW: Questionnaire of Planned Behavior on PAD–Walking
PNSES: Psychological Needs Satisfaction in Exercise Scale
BREQ-3: Behavioral Regulation in Exercise Questionnaire
IPAQ_E: International Physical Activity Questionnaire for the elderly
MVPA: Moderate-to-vigorous physical activity
SB: Sedentary behavior
HGS: Hand grip strength
GDS: Geriatric Depression Scale
GAS: Geriatric Anxiety Scale
MCID: Minimal clinically important difference
MID: Minimally important difference
SEM: Structural Equation Modeling

## References

[1] Nichols M, Townsend N, Scarborough P, Rayner M (2013). Cardiovascular disease in Europe: epidemiological update. Eur Heart J.

[2] Gerhard-Herman MD, Gornik HL, Barrett C, Barshes NR, Corriere MA, Drachman DE, Fleisher LA, Fowkes FG, Hamburg NM, Kinlay S, Lookstein R, Misra S, Mureebe L, Olin JW, Patel RA, Regensteiner JG, Schanzer A, Shishehbor MH, Stewart KJ, Treat-Jacobson D, Walsh ME (2017). 2016 AHA/ACC guideline on the management of patients with lower extremity peripheral artery disease: executive summary. Circulation..

[3] McDermott MM, Guralnik JM, Criqui MH, Liu K, Kibbe MR, Ferrucci L (2014). In Clinical Trials, Is the 6-minute walk test a better functional test of interventions for peripheral artery disease than treadmill walking tests?. Circulation..

[4] Mozaffarian D, Benjamin EJ, Go AS, Arnett DK, Blaha MJ, Cushman M (2016). Executive summary: heart disease and stroke statistics-2016 update. Circulation..

[5] McDermott M, Criqui M, Greenland P, Guralnik J, Liu K, Pearce W (2004). Leg strength in peripheral arterial disease: associations with disease severity and lower-extremity performance. J Vasc Surg.

[6] Kim M, Kim Y, Ryu GW, Choi M. Functional status and health-related quality of life in patients with peripheral artery disease: a cross-sectional study. Int J Environ Res Public Health. 2021;18(20). 10.3390/ijerph182010941. 10.3390/ijerph182010941 PMC8535998 34682683

[7] Aboyans V, Ricco J-B, Bartelink M-LEL, Björck M, Brodmann M, Cohnert T, Collet JP, Czerny M, de Carlo M, Debus S, Espinola-Klein C, Kahan T, Kownator S, Mazzolai L, Naylor AR, Roffi M, Röther J, Sprynger M, Tendera M, Tepe G, Venermo M, Vlachopoulos C, Desormais I, Widimsky P, Kolh P, Agewall S, Bueno H, Coca A, de Borst GJ, Delgado V, Dick F, Erol C, Ferrini M, Kakkos S, Katus HA, Knuuti J, Lindholt J, Mattle H, Pieniazek P, Piepoli MF, Scheinert D, Sievert H, Simpson I, Sulzenko J, Tamargo J, Tokgozoglu L, Torbicki A, Tsakountakis N, Tuñón J, de Ceniga MV, Windecker S, Zamorano JL, Windecker S, Aboyans V, Agewall S, Barbato E, Bueno H, Coca A, Collet JP, Coman IM, Dean V, Delgado V, Fitzsimons D, Gaemperli O, Hindricks G, Iung B, Juni P, Katus HA, Knuuti J, Lancellotti P, Leclercq C, McDonagh T, Piepoli MF, Ponikowski P, Richter DJ, Roffi M, Shlyakhto E, Simpson IA, Zamorano JL, Zelveian PH, Haumer M, Isachkin D, de Backer T, Dilic M, Petrov I, Kirhmajer MV, Karetova D, Prescott E, Soliman H, Paapstel A, Makinen K, Tosev S, Messas E, Pagava Z, Müller OJ, Naka KK, Járai Z, Gudjonsson T, Jonas M, Novo S, Ibrahimi P, Lunegova O, Dzerve V, Misonis N, Beissel J, Pllaha E, Taberkant M, Bakken T, Teles R, Lighezan D, Konradi A, Zavatta M, Madaric J, Fras Z, Melchor LS, Näslund U, Amann-Vesti B, Obiekezie A, ESC Scientific Document Group (2018). 2017 ESC Guidelines on the Diagnosis and Treatment of Peripheral Arterial Diseases, in collaboration with the European Society for Vascular Surgery (ESVS). Eur Heart J.

[8] Muluk SC, Muluk VS, Kelley ME, Whittle JC, Tierney JA, Webster MW, Makaroun MS (2001). Outcome events in patients with claudication: a 15-year study in 2777 patients. J Vasc Surg.

[9] Gardner A, Poehlman E (1995). Exercise rehabilitation programs for the treatment of claudication pain - a meta-analysis. JAMA.

[10] Sakamoto S, Yokoyama N, Tamori Y, Akutsu K, Hashimoto H, Takeshita S (2009). Patients with peripheral artery disease who complete 12-week supervised exercise training program show reduced cardiovascular mortality and morbidity. Circ J.

[11] Aquino R, Johnnides C, Makaroun M, Whittle JC, Muluk VS, Kelley ME, Muluk SC (2001). Natural history of claudication: long-term serial follow-up study of 1244 claudicants. J Vasc Surg.

[12] Norgren L, Hiatt WR, Dormandy JA, Nehler MR, Harris KA, Fowkes FGR (2007). Inter-society consensus for the management of peripheral arterial disease (TASC II). J Vasc Surg.

[13] Treat-Jacobson D, McDermott MM, Beckman JA, Burt MA, Creager MA, Ehrman JK, Gardner AW, Mays RJ, Regensteiner JG, Salisbury DL, Schorr EN, Walsh ME, American Heart Association Council on Peripheral Vascular Disease; Council on Cardiovascular and Stroke Nursing; Council on Epidemiology and Prevention; and Council on Lifestyle and Cardiometabolic Health (2019). Implementation of supervised exercise therapy for patients with symptomatic peripheral artery disease. Circulation..

[14] Harwood AE, Smith GE, Cayton T, Broadbent E, Chetter IC (2016). A systematic review of the uptake and adherence rates to supervised exercise programs in patients with intermittent claudication. Ann Vasc Surg.

[15] McDermott MM, Liu K, Guralnik JM, Criqui MH, Spring B, Tian L (2013). Home-based walking exercise intervention in peripheral artery disease: a randomized clinical trial. JAMA.

[16] McDermott MM, Guralnik JM, Criqui MH, Ferrucci L, Zhao L, Liu K, Domanchuk K, Spring B, Tian L, Kibbe M, Liao Y, Lloyd Jones D, Rejeski WJ (2014). Home-based walking exercise in peripheral artery disease: 12-month follow-up of the goals randomized trial. J Am Heart Assoc.

[17] McDermott MM, Spring B, Berger JS, Treat-Jacobson D, Conte MS, Creager MA (2018). Effect of a home-based exercise intervention of wearable technology and telephone coaching on walking performance in peripheral artery disease: the honor randomized clinical trial. JAMA.

[18] Hageman D, Fokkenrood HJP, Gommans LNM, van den Houten MML, Teijink JAW (2018). Supervised exercise therapy versus home-based exercise therapy versus walking advice for intermittent claudication (review). Cochrane Database Syst Rev.

[19] Golledge J, Singh TP, Alahakoon C, Pinchbeck J, Yip L, Moxon JV, Morris DR (2019). Meta-analysis of clinical trials examining the benefit of structured home exercise in patients with peripheral artery disease. Br J Surg.

[20] Room J, Hannink E, Dawes H, Barker K (2017). What interventions are used to improve exercise adherence in older people and what behavioural techniques are they based on? A systematic review. BMJ Open.

[21] Makris GC, Lattimer CR, Lavida A, Geroulakos G (2012). Availability of supervised exercise programs and the role of structured home-based exercise in peripheral arterial disease. Eur J Vasc Endovasc Surg.

[22] Galea MN, Weinman JA, White C, Bearne LM (2013). Do behaviour-change techniques contribute to the effectiveness of exercise therapy in patients with intermittent claudication? A systematic review. Eur J Vasc Endovasc Surg.

[23] Argent R, Daly A, Caulfield B (2018). Patient involvement with home-based exercise programs: can connected health interventions influence adherence?. JMIR mHealth uHealth.

[24] Khambati H, Boles K, Jetty P (2017). Google Maps offers a new way to evaluate claudication. J Vasc Surg.

[25] Fokkenrood HJP, Lauret GJ, Scheltinga MRM, Spreeuwenberg C, de Bie RA, Teijink JAW (2012). Multidisciplinary treatment for peripheral arterial occlusive disease and the role of eHealth and mHealth. J Multidiscip Healthc.

[26] Duscha BD, Piner LW, Patel MP, Crawford LE, Jones WS, Patel MR, Kraus WE (2018). Effects of a 12-week mHealth program on functional capacity and physical activity in patients with peripheral artery disease. Am J Cardiol.

[27] Mcdermott MM (2018). Exercise rehabilitation for peripheral artery disease: a review. J Cardiopulm Rehabil Prev.

[28] Joseph-Shehu EM, Ncama BP, Mooi N, Mashamba-Thompson TP (2019). The use of information and communication technologies to promote healthy lifestyle behaviour: a systematic scoping review. BMJ Open.

[29] Bickmore TW, Pusateri A, Kimani E, Paasche-Orlow MK, Trinh H, Magnani JW. Managing chronic conditions with a smartphone-based conversational virtual agent. IVA. 2018:119–24. 10.1145/3267851.3267908.

[30] Buinhas S, Claudio AP, Carmo MB, Balsa J, Cavaco A, Mendes A, García-Alonso J, Fonseca C (2019). Virtual assistant to improve self-care of older people with type 2 diabetes: first prototype. Gerontechnology.

[31] Lisetti C, Amini R, Yasavur U, Rishe N (2013). I can help you change! An empathic virtual agent delivers behavior change health interventions. ACM Trans Manag Inf Syst.

[32] Sillice MA, Morokoff PJ, Ferszt G, Bickmore T, Bock BC, Lantini R, Velicer WF (2018). Using relational agents to promote exercise and sun protection: assessment of participants’ experiences with two interventions. J Med Internet Res.

[33] Félix IB, Guerreiro MP, Cavaco A, Cláudio AP, Mendes A, Balsa J, Carmo MB, Pimenta N, Henriques A (2019). Development of a complex intervention to improve adherence to antidiabetic medication in older people using an anthropomorphic virtual assistant software. Front Pharmacol.

[34] Kruidenier LM, Nicolaï SPA, Willigendael EM, de Bie RA, Prins MH, Teijink JAW (2009). Functional claudication distance: a reliable and valid measurement to assess functional limitation in patients with intermittent claudication. BMC Cardiovasc Disord.

[35] Treat-Jacobson D, McDermott MM, Bronas UG, Campia U, Collins TC, Criqui MH, Gardner AW, Hiatt WR, Regensteiner JG, Rich K, American Heart Association Council on Peripheral Vascular Disease; Council on Quality of Care and Outcomes Research; and Council on Cardiovascular and Stroke Nursing (2019). Optimal exercise programs for patients with peripheral artery disease. Circulation..

[36] Gardner A, Skiner J (1991). Progressive vs single-stage treadmill tests for evaluation of claudication. Med Sci Sports Exerc.

[37] American College of Sports Medicine. Guidelines for exercise testing and prescription. 4th ed. Lea & Febiger. Philadelphia; 1991.

[38] American Thoracic Society (2002). American Thoracic Society ATS Statement: guidelines for the six-minute walk test. Am J Respir Crit Care Med.

[39] Ware JE, Sherbourne CD (1992). The MOS 36-item short-form health survey (Sf-36): I. conceptual framework and item selection. Med Care.

[40] Ferreira PL, Noronha Ferreira L, Nobre PL (2012). Physical and mental summary measures of health state for the Portuguese population. Rev Port Saude Publica.

[41] Nordanstig J, Wann-Hansson C, Karlsson J, Lundström M, Pettersson M, Morgan MBF (2014). Vascular Quality of Life Questionnaire-6 facilitates health-related quality of life assessment in peripheral arterial disease. J Vasc Surg.

[42] Larsen ASF, Reiersen AT, Jacobsen MB, Kløw NE, Nordanstig J, Morgan M, Wesche J (2017). Validation of the Vascular quality of life questionnaire-6 for clinical use in patients with lower limb peripheral arterial disease. Health Qual Life Outcomes.

[43] Correia M, Andrade-Lima A, Oliveira PL, Domiciano RM, Domingues WJ, Wolosker N (2018). Translation and validation of the Brazilian-Portuguese Short Version of Vascular Quality of Life Questionnaire in peripheral artery disease patients with intermittent claudication symptoms. Ann Vasc Surg.

[44] Nicolaï SPA, Kruidenier LM, Rouwet EV, Graffius K, Prins MH, Teijink JAW (2009). The walking impairment questionnaire: an effective tool to assess the effect of treatment in patients with intermittent claudication. J Vasc Surg.

[45] Ritti-Dias RM, Gobbo LA, Cucato GG, Wolosker N, Jacob Filho W, Santarém JM, Carvalho CR, Forjaz CL, Marucci Mde F (2009). Translation and validation of the walking impairment questionnaire in Brazilian subjects with intermittent claudication. Arq Bras Cardiol.

[46] Moss-Morris R, Weinman J, Petrie K, Horne R, Cameron L, Buick D (2002). The revised Illness Perception Questionnaire (IPQ-R). Psychol Health.

[47] Broadbent E, Petrie KJ, Main J, Weinman J (2006). The Brief Illness Perception Questionnaire. J Psychosom Res.

[48] Figueiras M, Marcelino DS, Claudino A, Cortes MA, Maroco J, Weinman J (2009). Patients’ illness schemata of hypertension: the role of beliefs for the choice of treatment. Psychol Health.

[49] Prochaska JO, DiClemente CC (1983). Stages and processes of self-change of smoking: toward an integrative model of change. J Consult Clin Psychol.

[50] Reed GR, Velicer WF, Prochaska JO, Rossi JS, Marcus BH (1997). What makes a good staging algorithm: examples from regular exercise. Am J Health Promot.

[51] Dannecker EA, Hausenblas HA, Connaughton DP, Lovins TR (2003). Validation of a stages of exercise change questionnaire. Res Q Exerc Sport.

[52] Hellsten L, Ann NC, Norman G, Burbank P, Braun L, Breger R (2008). Accumulation of behavioral validation evidence for physical activity stage of change. Health Psychol.

[53] Markland D (1999). Self-determination moderates the effects of perceived competence on intrinsic motivation in an exercise setting. J Sport Exerc Psychol.

[54] Silva MN, Vieira PN, Coutinho SR, Minderico CS, Matos MG, Sardinha LB, Teixeira PJ (2010). Using self-determination theory to promote physical activity and weight control: a randomized controlled trial in women. J Behav Med.

[55] Ferreira G, Pereira MG (2014). Validation of the questionnaire of planned behavior on diabetes: physical activity. Psicol Saúde e Doenças.

[56] Vlachopoulous S, Michailidou S (2009). Development and initial validation of a measure of autonomy, competence, and relatedness in exercise: the basic psychological needs in exercise scale. Meas Phys Educ Exerc Sci.

[57] Moutão JMRP, Serra LFC, Alves JAM, Leitão JC, Vlachopoulos SP (2012). Validation of the basic psychological needs in exercise scale in a Portuguese sample. Span J Psychol.

[58] Markland D, Tobin V (2004). A modification to the behavioural regulation in exercise questionnaire to include an assessment of amotivation. J Sport Exerc Psychol.

[59] Wilson PM, Rodgers WM, Loitz CC, Scime G (2007). “It’s Who I Am … Really!” The importance of integrated regulation in exercise contexts. J Appl Biobehav Res.

[60] Cid L, Monteiro D, Teixeira D, Teques P, Alves S, Moutão J, Silva M, Palmeira A (2018). The Behavioral Regulation in Exercise Questionnaire (BREQ-3) Portuguese-version: evidence of reliability, validity and invariance across gender. Front Psychol.

[61] Kim KS, Lee SJ, Suh JC (1975). Mini-Mental State: a practical method for grading the cognitive state of patients for the clinician. J Psychiatr Res.

[62] Santana I, Duro D, Lemos R, Costa V, Pereira M, Simões MR (2016). Mini-Mental State Examination: screening and diagnosis of cognitive decline, using new normative data. Acta Medica Port.

[63] Yesavage J, Rose T, Lum O (1983). Development and validation of a geriatric depression screening scale: a preliminary report. J Psychiatr Res.

[64] Santos AJ, Nunes B, Kislaya I, Gil AP, Ribeiro O (2019). Validation study of a reduced version of the Geriatric Depression Scale in Portugal. Análise Psicológica.

[65] Byrne GJ, Pachana NA (2011). Development and validation of a short form of the Geriatric Anxiety Inventory - the GAI-SF. Int Psychogeriatr.

[66] dos Silva L (2016). SV e., de Oliveira GM, Yokomizo JE, Saran LF, Bottino CM de C, Yassuda MS. The geriatric anxiety inventory in primary care: applicability and psychometric characteristics of the original and short form. Arch Clin. Psychiatry..

[67] International physical Activity Questionaire [Internet]. 2005 [cited 2021 May 20]. Available from: https://sites.google.com/site/theipaq/

[68] Hurtig-Wennlf A, Hagstrmer M, Olsson LA (2010). The International Physical Activity Questionnaire modified for the elderly: aspects of validity and feasibility. Public Health Nutr.

[69] Teixeira PJ, Marques A, Lopes C, Sardinha LB, Mota JA (2019). Prevalence and preferences of self-reported physical activity and nonsedentary behaviors in Portuguese adults. J Phys Act Health.

[70] Bohannon RW, Magasi S (2015). Identification of dynapenia in older adults through the use of grip strength t-scores. Muscle Nerve.

[71] Van Den Houten MML, Gommans LNM, Van Der Wees PJ, Teijink JAW (2016). Minimally important difference of the absolute and functional claudication distance in patients with intermittent claudication. Eur J Vasc Endovasc Surg.

